# Developing a national dental education research strategy: priorities, barriers and enablers

**DOI:** 10.1136/bmjopen-2016-013129

**Published:** 2017-03-29

**Authors:** Rola Ajjawi, Karen L Barton, Ashley A Dennis, Charlotte E Rees

**Affiliations:** 1 Centre for Assessment in Research and Digital Learning, Deakin University, Geelong, Victoria, Australia; 2 Division of Food and Drink, School of Science, Engineering & Technology, Abertay University, Dundee, UK; 3 Centre for Medical Education, University of Dundee, Dundee, UK; 4 Faculty of Medicine, Nursing & Health Sciences, Monash University, Melbourne, Victoria, Australia

**Keywords:** dental education research, priority setting, online questionnaire, EDUCATION & TRAINING (see Medical Education & Training), STATISTICS & RESEARCH METHODS

## Abstract

**Objectives:**

This study aimed to identify national dental education research (DER) priorities for the next 3–5 years and to identify barriers and enablers to DER.

**Setting:**

Scotland.

**Participants:**

In this two-stage online questionnaire study, we collected data with multiple dental professions (eg, dentistry, dental nursing and dental hygiene) and stakeholder groups (eg, learners, clinicians, educators, managers, researchers and academics). Eighty-five participants completed the Stage 1 qualitative questionnaire and 649 participants the Stage 2 quantitative questionnaire.

**Results:**

Eight themes were identified at Stage 1. Of the 24 DER priorities identified, the top three were: role of assessments in identifying competence; undergraduate curriculum prepares for practice and promoting teamwork. Following exploratory factor analysis, the 24 items loaded onto four factors: teamwork and professionalism, measuring and enhancing performance, dental workforce issues and curriculum integration and innovation. Barriers and enablers existed at multiple levels: individual, interpersonal, institutional structures and cultures and technology.

**Conclusions:**

This priority setting exercise provides a necessary first step to developing a national DER strategy capturing multiple perspectives. Promoting DER requires improved resourcing alongside efforts to overcome peer stigma and lack of valuing and motivation.

**Strengths and limitations of this study**

- Exploratory factor analysis enabled identification of key priority areas for dental education research with representation from multiple stakeholders enabling less dominant voices to be incorporated.
- The two-stage online questionnaire approach promotes transparency of the provenance of priorities and identification of barriers and enablers that can be harnessed in a research strategy.
- It was not possible to calculate a response rate for Stage 2 but a large and broad sample of dental education stakeholders across institutions and regions in one country participated.
- Participant sample characteristics varied from Stage 1 to Stage 2; to overcome this potential sample bias, the Stage 2 questionnaire included open-ended questions where respondents could add new priorities, barriers and enablers not identified in Stage 1.

## Introduction

Having an explicit research strategy, against which research gains may be measured, is one of the markers of a ‘vital and sustainable’ research environment as stipulated by the UK Research Excellence Framework 2014.^1^ Indeed, Chalmers and Glasziou^2^ have estimated that up to 85% of research investment is wasted because of low-priority research questions that do not meet stakeholder needs. In order to reduce such waste, there is a call for improving the transparency of processes by which priorities are set, making clear how they take account of the needs of potential users of research.^3^ Better prioritisation of future research is necessary to increase research value in a context of limited human and monetary resources.^4^ It is also argued that prioritisation of research is essential for a profession to systematically advance its scientific base and stimulate national research efforts.^5^ While various priority-setting exercises (PSEs) have been published for medical education research (MER) across numerous countries^6–8^ and for primary dental research,^5^ ^9^ ^10^ to the best of our knowledge, none have been published for dental education research (DER). The current study aims to address this gap in the DER literature.

### Dental education priorities

In one European context—Scotland—the 2010 Strategy for Oral Health Research recommended the need for a DER strand,^11^ leading to the formation of the Dental Education Research Group (DERG), with representation from dental and dental care professional schools across Scotland and National Health Service Education for Scotland (NES). A stated aim of this group was to develop a national DER strategy for Scotland. While no published literature on DER priorities could be identified, three were found which related to primary dental care^9^ and dental hygiene research.^5^ ^10^

Using a Delphi technique with an expert group (undisclosed sample size) including various stakeholders (eg, general dental practitioners, academics, executives from health authorities, members of patient advisory groups, specialists, consultants in dental public health, the British Dental Association and the UK Faculty of General Dental Practice), Palmer and Batchelor^9^ invited Delphi group members to submit their perceived five key priorities for research in primary dental care. The resulting list contained 36 priority topics grouped into three main categories: clinical, patient centred and the dental team. These 36 items were then ranked by the participants and consensus was reached following two rounds of the Delphi process for five key primary dental care research areas including: evaluation of the costs and benefits of whole team training. While this theme relates to DER, the authors provided little explanation of what this theme involved. It is interesting to note that the 10^th^-ranked priority was related to ‘education and training needs in primary dental care’, but this was not elaborated on either.

Also using a Delphi technique, this time with 49 dental hygiene experts and key opinion leaders, Forrest and Spolarich^5^ updated the American Dental Hygienists' Association (ADHA) National Dental Hygiene Research Agenda (NDHRA), originally developed in 1995.^10^ Using the same approach and sample size as the original study, with good return rates and internal consistency recorded for their two rounds of Delphi, consensus was reached on 42 items (five more items than in 1995). The 42 items were grouped into five broad categories, one of which, the ‘professional education and development’ category referred to: ‘educational methods, curricula, students and faculty; recruitment and retention of students and faculty; and promoting graduate education and career path options’.^5^

Focusing on the professional education and development theme, table 1 shows the priority items related to this theme from 1995 and 2009, respectively. The most recent items have a broader focus which include research on the achievement of student learning outcomes, as well as research on effective curricular models, educational processes, promoting research and researching faculty. This highlights greater sophistication and granularity within the more recent dental hygiene education research items. However, the study is specifically for dental hygienists in the USA (with only one stakeholder group's voice represented in the data). All three Delphi studies highlighted above did not specifically focus on DER, although they did identify some DER items, and ranged widely in methodological rigour (including inadequate explanation of sample size and characteristics and limited to a single disciplinary group).

**Table 1:** Comparison of the American Dental Hygienists' Association priority items listed for the ‘Professional Education and Development’ theme^5^ ^10^

Forrest *et al* (1995)^10^	Forrest and Spolarich (2009)^5^
Investigate the extent to which new research findings are incorporated into the dental hygiene curriculum. Investigate the extent to which students are taught critical thinking and decision-making skills. Identify the factors leading to curriculum modification and reform in dental hygiene academic programmes. Investigate the extent to which students are taught self-assessment and evaluation skills. Develop a predictive model for future needs/demands for dental hygiene personnel.	Investigate the extent to which new research findings are incorporated into the dental hygiene curriculum. Validate and test measures that evaluate student critical thinking and decision-making skills. Evaluate the extent to which current dental hygiene curriculum prepares dental hygienists to meet the increasingly complex oral health needs of the public. Critically appraise current methods of evaluating (assessing) clinical competency. Investigate how other health professions have established the masters and doctoral levels of education as their entry level into practice. Identify the factors that affect recruitment and retention of faculty. Assess how educators are socialising students into research. Investigate curriculum models for training and certification of competency in specialty areas.

### Medical education priorities

Turning to the medical education literature, we identified three PSEs conducted either in New Zealand, Canada or the UK. In New Zealand, through a modified Delphi approach with 17 medical educators in the first round and 11 in the second round, researchers identified the following MER priorities: engaging in community and clinical learning environments; improving recruitment and retention; assessing professional behaviours; promoting quality feedback; engaging clinical teachers and improving phases of transition.^8^ In Canada, 30 key informants were interviewed (eg, academic leaders in various healthcare professions, leaders of healthcare and trainee institutions or programmes, health or education ministry officials, representatives of medical professional organisations or journals and members of the public) and the data analysed alongside a literature review.^7^ They identified the following 10 medical education priorities: address individual and community needs; enhance admissions processes; build on the scientific basis of medicine; promote prevention and public health; address the hidden curriculum; diversify learning contexts; value generalism; advance interprofessional and intraprofessional practice; adopt a competency-based and flexible approach and foster medical leadership. Both these studies do not identify how the stakeholders' views varied.

In 2014, two of the authors of the current study (AD and CER) published the findings of a two-stage PSE for MER in Scotland.^6^ Stage 1 involved a qualitative questionnaire seeking priorities for MER from multiple stakeholders (including learners, educators, leaders and patient representatives) and why these were perceived as priorities. Stage 2 involved a quantitative questionnaire to rank the 21 priorities identified in Stage 1. The top ranked priorities were: (1) balancing conflicts between service and training; (2) providing useful feedback; (3) promoting resiliency and well-being; (4) creating an effective workplace learning culture; (5) selecting and recruiting doctors to reflect need and (6) ensuring curricula prepare trainees for practice. Using factor analysis, we identified five key thematic priorities: (1) culture of learning together in the workplace; (2) enhancing and valuing the role of educators; (3) curriculum integration and innovation; (4) bridging the gap between assessment and feedback and (5) building a resilient workforce. Furthermore, participants explained why they chose these priorities: patient safety; quality of care; investing for the future; policy and political agendas and evidence-based education. Importantly, we found significant differences in the priorities among different stakeholders with patients, for example, rating the culture of learning together in the workplace as more important that non-patients.^6^ This highlights the need to involve multiple stakeholders in PSEs including taking into account the views of patients.^12^ It is unclear how these priorities apply to DER, however, given differences in disciplinary roles and cultures between medicine and dentistry. It also remains unclear what barriers and enablers exist to the conduct of DER, which might undermine the success of such strategic documents.

### Barriers and enablers to DER and MER

Research by Blinkhorn *et al*^13^ with 81 (of 91) dentists attending research training identified the following barriers to undertaking research in primary dental care: lack of incentives to undertake research, lack of research skills, isolation of dental practitioners, lack of research time, fear that research would generate paperwork and lack of support. The large number of participants not attending the research training (872 dentists were invited) may be in part due to lack of interest in research. Provision of rewards (financial or qualification) and linkages with academic institutions were seen as enablers. Research by Jowett *et al*^14^ with UK general practitioners (n=1351; 49% response rate) identified lack of time (92%), lack of staff to collect data (73%) and lack of funding (71%). In all, 41% of respondents reported no interest in research. In another study exploring barriers and enablers to primary healthcare research, more than half of participants (59%) identified time as the biggest barrier, followed by financial constraints (38%) and limited support in the workplace (12%).^15^ Common enablers were collaboration in research teams, access to academic mentors and acquiring research skills.^15^ The study by Hodges *et al*^7^ already discussed identified five enablers of medical education practice and research in Canada: realigning accreditation standards, building capacity for change, increasing national collaboration, improving the use of technology and enhancing faculty staff development. So, while various researchers in healthcare have begun to outline the barriers and enablers of healthcare and healthcare education research, to the best of our knowledge, none have specifically explored the barriers and enablers of DER which might affect the success of DER strategies.

### Rationale and aim

In looking at education research priorities across dentistry and medicine, while some priorities are similar (eg, effectiveness of curriculum models), there are also many notable differences (eg, focus on basic sciences in one MER PSE, inculcating into research practices in one dental PSE). This likely reflects differences between DER and MER. For example, dental students undertake invasive and irreversible procedures; tend to be more hands-on with procedural skills than medical students at the undergraduate level and carry higher levels of responsibility as full-fledged professionals on graduation, with less emphasis on postgraduate training.^16^ ^17^ In addition, differences might also relate to the countries in which PSEs are conducted. There seems to be a need to drive forward systematic and strategic research at the national level to enrich disciplinary knowledge in dental education. A content analysis of all papers published in the two leading DER journals (2003–2008) found that the majority of papers were descriptive and focused on local curriculum evaluation initiatives.^18^ Furthermore, little is known about specific barriers and enablers in DER. Therefore, in this study, we sought to answer the following research questions:

1. What are the top DER priorities for Scotland for the next 3–5 years according to multiple stakeholders?
2. What differences (if any) exist between the priorities identified by different types of stakeholders?
3. What are multiple stakeholders' perceptions of the barriers and enablers to DER?

## Methods

### Study design

In the current study, we used a similar methodological approach to our earlier MER PSE.^6^ We chose this multistaged questionnaire approach because it accounted for multiple stakeholders through inclusive recruitment and data collection and analysis approaches; hence, preventing the interests of one group from dominating over others. Choosing the same methods also enabled us to compare the MER and DER priorities for one country.

### Questionnaire design

A two-staged online questionnaire study using Bristol Online Survey (https://www.onlinesurveys.ac.uk/) was conducted. The Stage 1 qualitative online questionnaire was adapted from our previous study.^6^ It contained open-ended questions, which asked participants what they thought the top three DER priorities in Scotland were over the next 3–5 years and why these were perceived to be the top priorities. In contrast to our previous MER study, it also asked participants about perceived barriers and enablers to DER. The questionnaire was open-ended in the hope that participants would identify a broad range of DER priorities.

The Stage 2, quantitative online questionnaire was developed from the Stage 1 findings and included 24 items. Of these 24 items, 19 items were identified from the Stage 1 DER PSE (15 items were as per the MER PSE, four were new items). Additionally, in order to enable better comparisons to be made with our MER PSE,^6^ we added the five items from the MER PSE (not found in our Stage 1 dental PSE) but we tailored these specifically to dentistry. We thought that if these items were genuinely not priorities for DER, then Stage 2 participants would rank them as being less important.

The Stage 2 questionnaire asked participants to rate the importance of each item on a 6-point Likert scale (1= not important, 6= very important). It then asked participants to identify their top five priorities out of the list of 24 topics and to state why they chose these items as priorities and to list any additional priorities not included in the list. Finally, participants were asked to choose the top three perceived barriers and enablers to DER identified in the Stage 1 questionnaire. Participants were also able to include additional barriers or enablers under the heading ‘other’ with free text responses.

Both questionnaires also included a series of questions with regards to participant demographic (eg, age, gender and ethnicity) and professional (eg, stakeholder group and region) characteristics. The questionnaires were checked and piloted by three members of the DERG to ascertain ease of comprehension and length of completion prior to being launched with minor amendments to the wording of questions (eg, inclusion of the term ‘educator’ rather than ‘trainer’).

### Sampling and recruitment

Maximum variation sampling^19^ was used for both stages as it was recognised that different stakeholder groups may have differing perspectives with regards to priorities for DER.^6^ The research team, in collaboration with DERG, identified a named lead for each region of Scotland involved in dental education. DERG helped identify key stakeholder groups across Scotland, including individuals from urban, rural and remote settings. This inclusive focus across stakeholder groups and regions was important to gain a breadth of perspectives and is in accordance with recommendations for research priority setting in the literature.^6^ ^12^

For Stage 1, DERG members and the named leads for each region nominated individuals (within each of the stakeholder groups) whom they felt had sufficient knowledge of DER to answer open-ended questions about priorities, barriers and enablers. Individual invitation emails were sent to a total of 357 stakeholders (sometimes at different times) across the 3.5-month data collection period. The invite emails included the information sheet and link to the online questionnaire. Reminder emails were sent 2 and 3 weeks post initial invitation. Participants completed the questionnaire between June and September 2014 due to the staggered nature of recruitment (eg, if we had no participants representing a particular stakeholder group, we would invite new participants representing that group). Recruitment efforts continued until each of the broad stakeholder groups were represented with at least one participant in each group.

For Stage 2, in order to maximise recruitment, multiple recruitment methods were used. These included: (1) emails sent from academic leads at each institution, from the Scottish Dental Practice Based Research Network and to individuals on the Scottish dental-related NES email lists; (2) posters and flyers at each academic institution (including a link to the online questionnaire); (3) personal endorsements from institutional leads at lectures, meetings and training sessions; (4) flyers sent to all dental practices in Scotland and an article in the NHS ‘Mouthpiece Extra’ newsletter; (5) information on the Centre for Medical Education, Scottish Dental and Scottish Oral Health Collaboration websites and (6) snowballing though individuals working in dentistry or dental care professionals. Additionally, individuals who were invited to complete the Stage 1 questionnaire were also invited to complete the Stage 2 questionnaire. Participants completed the questionnaire over a period of 3 months between October 2014 and January 2015. As with Stage 1, recruitment was staggered and reminder invitations were sent at 2 and 3 weeks post initial invitation where possible and recruitment continued until it was felt that we had exhausted all feasible routes.

### Data analysis

Qualitative and quantitative data analyses were conducted. Descriptive statistics were used to identify the characteristics of the samples using IBM SPSS Statistics V.21.0 (IBM Corporation, USA).

Thematic framework analysis was conducted for the qualitative data collected from the Stage 1 questionnaire.^20^ The thematic analysis allowed the key themes in the data to be identified around the issues perceived by stakeholders as representing the top priorities in DER, as well as coding the barriers and enablers. A sample of 50 questionnaires (of the 85 returned; 59%) was analysed by four researchers (two researchers analysing 25 each) in order to develop the preliminary coding framework. The development of this coding framework was inductive (RA and KLB analysed their 25 questionnaires without in-depth knowledge of the original coding framework) and deductive (AAD and CER analysed their 25 questionnaire mindful of their previously developed coding framework).^6^ We used Atlas-ti qualitative data analysis software V.7 (GmbH, Berlin) to code and interrogate all qualitative data from both questionnaires in order to establish barriers and enablers and similarities and differences in the priorities identified by the different stakeholder groups. Responses coded as ‘other’ were examined by two of the researchers (RA and KLB) and where appropriate recategorised.

For the quantitative data collected in the second questionnaire, median and interquartile range (IQR) were calculated to identify Likert scale ratings of importance for each of the 24 topics. In order to explore the ‘top five’ topics, ranked scores were calculated as follows: where the participant had rated a topic as having first priority, it was given 5 points; topics rated as being second, third, fourth and fifth were given 4, 3, 2 and 1 points, respectively. The scores given to each of the 24 items by all participants were then summated to identify scores and rankings of importance for each item. Participants were asked to choose their top three perceived barriers and enablers provided as options based on the coding from Stage 1; these were simply counted.

Similarities and differences in participants' identified priorities across the stakeholder groups were examined using exploratory factor analysis (EFA) in order to reduce the data set but retain as much of the original information as possible. Confirmatory factor analysis was not employed as the 24 items in the DER questionnaire differed from the 21 MER items identified in our earlier study. EFA was conducted on the importance ratings of the 24 items, using principal components analysis with direct oblimin rotation, to identify higher order factors. A total score was then calculated for each participant for each factor. As each item could be scored 1–6, total factor scores depended on how many items loaded on each factor (F1=8 items, scores of 8–48; F2=5 items, scores of 5–30; F3=7 items, scores of 7–42; and F4=5 items, scores of 5–30). Kruskal-Wallis and Mann-Whitney tests were used to establish any significant differences in factor scores across demographics and professional roles. We determined internal consistency of the factors using Cronbach's α.

## Results

### Participant characteristics

Of the 356 individuals invited at Stage 1, 85 (24%) completed the qualitative survey, each identifying at least one priority. The highest proportion of respondents was men (n=46, 54%), aged 50–59 years (n=30, 35%) and white (n=81, 95%). All stakeholder groups were represented, as were regions (including individuals in urban, rural and remote settings), NES, each of the four Scottish dental schools and educational institutions responsible for teaching other members of the dental team (eg, dental nurses, dental hygienists) and a few individuals with national or international dental education and research roles (see table 2).

**Table 2:** Characteristics of respondents to both online questionnaires

Characteristic	Stage 1 (n, (%)) N=85	Stage 2 (n, (%)) N=649
Age, years (%)		
≤20	0	19 (2.9)
20–29	8 (9.4)	219 (33.7)
30–39	13 (15.3)	124 (19.1)
40–49	26 (30.6)	129 (19.9)
50–59	30 (35.3)	136 (21.0)
60–69	8 (9.4)	21 (3.2)
≥70	0	1 (0.2)
Gender (%)		
Male	46 (54.1)	242 (37.3)
Female	39 (45.9)	407 (62.7)
Ethnicity (%)		
White, White Scottish, White British	81 (95.3)	565 (87.1)
Non-White*	4 (4.7)	84 (12.9)
Stakeholder group† (%)		
Learners	9 (10.6)	189 (29.1)
Educators	74 (87.1)	189 (29.1)
Dentists	53 (62.4)	581 (89.5)
Dental care professionals	16 (18.8)	111 (17.1)
Researchers	12 (14.1)	26 (4.0)
Patient representatives	1 (1.2)	2 (0.3)
Region* (%)		
East	37 (43.5)	185 (28.5)
North	20 (23.5)	89 (13.7)
South East	8 (9.4)	84 (12.9)
West	19 (22.4)	267 (41.1)
National (Scotland)	9 (10.6)	32 (4.9)
UK	3 (3.5)	13 (2.0)
International	0	4 (0.6)

*Non-White is composed of a heterogeneous grouping of ethnicity in order to maintain anonymity.

†Unless indicated, any percentages that do not add up to 100% are due to rounding; Percentages add up to more than 100% because participants sometimes belonged to multiple groups.

For the Stage 2 questionnaire, 649 individuals participated. It was not possible to calculate a response rate for this stage because we do not know how many individuals received the invitation, but the sample was diverse and again included all stakeholder groups, regions, NES, each of the four dental schools and other educational institutions in Scotland. The majority of respondents in Stage 2 were women (n=407, 63%), aged 20–29 years (n=219, 33.7%) and white (n=565, 87%) (table 2).

### RQ1: What are the top DER priorities for Scotland for the next 3–5 years?

Eight key themes were identified as a result of the Stage 1 framework analysis: issues pertaining to the learner; issues pertaining to the educator; working with others in the workplace; workplace culture; curriculum integration; curriculum content; curriculum delivery and assessment/feedback. (The online supplementary file provides a breakdown of each of these themes, subthemes (items), definitions and illustrative quotes). The subthemes below formed the items for the Stage 2 questionnaire. (Note that we use the term ‘educator’ to refer to ‘educators’, ‘tutors’, ‘preceptors’ and ‘trainers’ at undergraduate and postgraduate levels and the term ‘learner’ to refer to learners and trainees at undergraduate and postgraduate levels, respectively).

10.1136/bmjopen-2016-013129.supp1 supplementary file

The two different indicators of perceived priority in the Stage 2 questionnaire (ie, the median and IQR of each Likert scale response for the 24 items and the summation of the rankings for the items identified in the ‘top five’) are presented in table 3, in descending order of the total rank score. Two topics were rated most highly according to both indicators: (1) role of assessments in identifying competence (item 1), and (2) undergraduate curriculum prepares for practice (item 15). Four further topics were also rated highly by both indicators: promote teamwork within the dental team (item 12), role of assessments in identifying underperformance (item 3), providing useful feedback (item 2) and enhancing communication skills (item 21).

**Table 3:** The importance of each topic based on two indicators of perceived priority (higher scores on each indicator reflect greater perceived priority)

Item		Score, median (IQR)	Total rank score (overall ranking)
1	Role of assessments in identifying competence	5 (4–6)	1042 (1)
15	Undergraduate curriculum prepares for practice	5 (5–6)	1021 (2)
12	Promote teamwork within the dental team	5 (4–6)	687 (3)
3	Role of assessments in identifying underperformance	5 (4–6)	679 (4)
2	Providing useful feedback	5 (4–6)	676 (5)
21	Enhance communication skills	5 (4–6)	631 (6)
24	Teaching evidence-based practice	5 (4–6)	505 (7)
*7	Select/approve educators	5 (4–6)	452 (8)
11	Effective workplace learning culture	5 (4–6)	418 (9)
4	Select/recruit dental professionals	5 (4–6)	383 (10=)
23	Tailoring teaching to individual learning needs	5 (4–6)	383 (10=)
*6	Resiliency/well-being	5 (4–6)	366 (12)
13	Foster interprofessionalism	5 (4–6)	323 (13)
22	Professionalism	5 (4–6)	318 (14)
*8	Support/value role of educators	5 (4–6)	288 (15)
10	Balance education/service conflicts	5 (4–5)	282 (16)
20	Role of simulation in education	5 (4–6)	258 (17)
19	Impact of technology	4 (4–5)	216 (18)
16	Postgraduate curriculum prepares for practice	5 (4–6)	179 (19)
*14	Develop leadership	4 (4–5)	152 (20)
9	Faculty development	5 (4–5)	150 (21)
*5	Career choice	4 (3–5)	126 (22)
18	Vertically integrate undergraduate/postgraduate curricula	4 (3–5)	121 (23)
17	Role of formal/informal curricula	4 (3–5)	52 (24)

*Items 5, 6, 7, 8, 14 were not identified in Stage 1 dental education research (DER) priority-setting exercise (PSE) but were included in Stage 2 based on the medical education research (MER) PSE.^6^

Participants explained why they chose these priorities around seven themes: patient safety; quality of care; investing for the future; policy and political agendas; evidence-based education; improving student learning and personal interest. The most common reasons given for rating the above six priorities as most important are as follows: (1) role of assessments in identifying competence (ensuring patient safety, promoting student learning and developing an evidence base to ensure that the assessments are robust and trustworthy); (2) undergraduate curriculum prepares for practice (investing in the future dental workforce, promoting student learning and delivering evidence to improve curriculum content, design and delivery for real-world practice); (3) promote teamwork within the dental team (ensuring patient safety and quality of care); (4) role of assessments in identifying underperformance (ensuring patient safety and quality of care standards, and promoting student learning through early detection and remediation); (5) providing useful feedback (promoting student learning and ensuring quality of care) and (6) enhancing communication skills (ensuring quality of care and patient safety through good communication with patients and colleagues and promoting student learning).

### RQ2: What are the relationships among the priorities identified and the stakeholder groups?

This section showcases patterns of responses among priorities and also among different stakeholders. EFA was conducted to identify the factors underpinning the 24 items in Stage 2 and to examine differences between participant groups in a quantitative manner. The Kaiser-Meyer-Olkin measure of sampling adequacy of 0.93 verified that the sampling adequacy for the analysis was well above the acceptable limit of 0.5. (The Kaiser-Meyer-Olkin statistic varies from 0 to 1 with a value close to 1 indicating the patterns of correlations are relatively compact and that the factor analysis should yield distinct and reliable factors.^21^) Bartlett's test of sphericity demonstrated that the correlations among the items were sufficient (χ2 (210)=8275.90, p<0.001).

Following EFA with three-factor, four-factor and five-factor solutions, the research team decided that the four-factor solution made the most theoretical sense after discussing each solution in depth. This solution included all 24 items in the factors and had minimal overlap of the items across multiple factors (note that only one item loaded onto more than one factor: item 16 loaded onto factors 2 and 4 but this overlap made theoretical sense). The four factors met Kaiser's criterion with eigenvalues of >1 and together explained 58.83% of the variance. Table 4 presents the results of the factor analysis and provides the factor loadings after rotation for each of the four factors. The cut-off for inclusion of a variable for interpretation of a factor was 0.33. While 0.32 equates to ∼10% overlapping variance with other items in that factor^22^ and was used by Dennis et al,^6^ one item that was 0.32 did not make theoretical sense in relation to the factor it loaded on, hence the decision to use 0.33 in this current study. The items that clustered on each factor suggested that factor 1 focuses on teamwork and professionalism, factor 2 on measuring and enhancing performance, factor 3 on dental workforce issues and factor 4 on curriculum integration and innovation.

**Table 4:** Pattern matrix from exploratory factor analysis

		Component			
Item	Description of item	F1	F2	F3	F4
12.	Promote teamwork within the dental team	0.865			
13.	Foster interprofessionalism	0.826			
21.	Enhance communication skills	0.679			
24.	Teaching evidence-based practice	0.636			
22.	Professionalism	0.584			
14.	Develop leadership	0.523			
11.	Effective workplace learning culture	0.508			
23.	Tailoring teaching to individual learning needs	0.464			
1.	Role of assessments in identifying competence		0.866		
3.	Role of assessments in identifying underperformance		0.795		
2.	Providing useful feedback		0.521		
15.	Undergraduate curriculum prepares for practice		0.472		
7.	Select/approve educators			−0.817	
8.	Support/value role of educators			−0.759	
9.	Faculty development			−0.654	
6.	Resiliency/well-being			−0.615	
5.	Career choice			−0.610	
10.	Balance education/service conflicts			−0.602	
4.	Select/recruit dental professionals			−0.588	
18.	Vertically integrate undergraduate/postgraduate curricula				0.732
19.	Impact of technology				0.711
17.	Role of formal/informal curricula				0.700
20.	Role of simulation in education				0.608
16.	Postgraduate curriculum prepares for practice		0.331		0.415
	Eigenvalues	9.88	1.69	1.37	1.20
	% of variance	41.17	7.04	5.73	4.98
	α-value	0.89	0.79	0.86	0.81

F1, Teamwork and professionalism; F2, Measuring and enhancing performance; F3, Dental workforce issues; F4, Curriculum integration and innovation.

### Gender

Significant differences were found between genders for factors 1 (teamwork and professionalism) and 3 (dental workforce issues) with women rating these factors more highly than men (table 5).

**Table 5:** Relationship between participant characteristics and ratings of importance

Variable*	Group	Median (IQR)	Test statistics†
Factor 1	Male	36 (32–41)	Z=−5.03, p<0.001, r=−0.20
	Female	40 (35–43)	
Factor 3	Male	31 (25–35)	Z=−4.03, p<0.001, r=−0.16
	Female	33 (29–36)	
Factor 1	White	38 (33.75–42)	Z=−3.98, p=<0.001, r=−0.16
	Non-White	41 (37–45)	
Factor 2	White	25 (22–27)	Z=−3.60, p<0.001, r=−0.14
	Non-White	27 (24–29)	
Factor 3	White	32 (27–35)	Z=−3.45, p<0.001, r=−0.14
	Non-White	35 (29–38)	
Factor 4	White	22 (18–24)	Z=−5.61, p<0.001, r=−0.22
	Non-White	25 (22–27)	
Factor 2	18–39	25 (23–28)	X2=6.97, d.f.=2, p=0.031, φc=0.07
	40–59	24 (20.5–26)	
	60+	26 (23–28)	
Factor 1	Dentist	37 (32–42)	Z=−6.74, p<0.001, r=−0.27
	Non-dentist	41 (37–45)	
	Dental care professional	41 (37–45)	Z=−6.55, p<0.001, r=−0.26
	Non-dental care professional	37 (33–42)	
Factor 3	Dentist	31 (26–35)	Z=−5.55, p<0.001, r=−0.22
	Non-dentist	34 (30.25–37)	
	Dental care professional	34 (31–38)	Z=−5.58, p<0.001, r=−0.22
	Non-dental care professional	31 (26–35)	
Factor 4	Dentist	22 (18–24)	Z=−4.13, p<0.001, r=−0.16
	Non-dentist	23 (20–26)	
	Dental care professional	23 (21–26)	Z=−4.03, p<0.001, r=−0.16
	Non-dental care professional	22 (18–24)	

*Factor 1= teamwork and professionalism; factor 2= measuring and enhancing performance; factor 3= dental workforce issues; factor 4= curriculum integration and innovation.

†Test statistics are Mann-Whitney Z-scores, p value, effect size r or Kruskal-Wallis χ^2^ value with d.f., p value, effect size Φc. For the Mann-Whitney test, effect sizes for significant findings were calculated as: r=Z/√n. Magnitudes of effect sizes for Cohen's r are: 0.1: small; 0.3: medium and 0.5: large.^23^ For the Kruskal-Wallis test, effect sizes were calculated using Cramer's V Φc=√(χ^2^/[n{k−1}]), where k equals the smaller of rows or columns. Magnitudes of effect sizes for Cramer's phi are: 0.1 to <0.2: weak association; 0.2 to <0.4: moderate association; 0.4 to <0.6: relatively strong association and ≥0.6: strong association.^24^

### Ethnicity

Significant differences were found between white and non-white groups for all factors with non-white participants rating them all more highly than white participants (table 5). Note that there were insufficient individuals within every ethnic group for more extensive statistical analyses.

### Age

Differences in scores for different age groups across the factors, assessed using Kruskal-Wallis tests, suggested that factor 2 (measuring and enhancing performance) scored significantly differently across the three age ranges (table 5). Follow-up Mann-Whitney tests suggested that individuals aged ≥60 years rated factor 2 more highly than those aged 18–39 years (Z=−2.07, p<0.039, r=−0.11; small effect) and those aged 40–59 years (Z=−2.34, p<0.019, r=-0.14; small effect).

### Region

No significant differences were found among responses given by participants from any of the regions (East, North, South East and West).

### Professional role

There were no differences found for factor scores between learners and non-learners; educators and non-educators or researcher and non-researchers. However, non-dentists (this primarily includes dental care professionals and administrators and researchers) rated factors 1 (teamwork and professionalism), 3 (dental workforce issues) and 4 (curriculum integration and innovation) more highly than dentists. Also, dental care professionals (not including dentists) rated factors 1 (teamwork and professionalism), 3 (dental workforce issues) and 4 (curriculum integration and innovation) more highly than non-dental care professionals with small to medium effect sizes (see table 5).

### RQ3: What are the main barriers and enablers to DER?

Overall barriers and enablers existed at the level of the individual, interpersonal relationships, institutional structures and cultures and technology. The top five perceived barriers to DER (including illustrative quotes) were lack of: time ‘Time pressure for clinicians who are not full-time academics’ (institutional structures and cultures; n=355; 55%), external funding: ‘Funding for educational research’ (institutional structures and cultures; n=203; 31%), staff motivation: ‘Apathy’ (individual; n=198; 31%), valuing of DER by individuals: ‘Clinicians do not perceive education research to be valuable’ (individual; n=157; 24%) and resources and infrastructure: ‘Limited expertise in educational theory and qualitative research methods. Relates to resource issue’ (institutional structures and cultures; n=150; 23%).

The top five perceived enablers to DER were: staff motivation: ‘There are many enthusiastic educators who could be encouraged to report their work’ (individual; n=276; 43%), sufficient time: ‘Providing protected time’ (institutional structures and cultures; n=209; 32%), valuing of DER by individuals: ‘Staff recognition of the need to undertake good quality work in this regard’ (individual; n=168; 26%), staff expertise in dental education: ‘We need to address the skills gap in dental educational research. The will is there but it needs nurturing and development’ (individual; n=165; 25%) and availability of external funding: ‘Provision of pump-priming funds’ (institutional structures and cultures; n=149; 23%). Interestingly, valuing of DER by institutions was listed as a barrier (‘Universities do not rate research in medical/dental education as highly as that in clinical/bioscience research’) and enabler (‘Opportunities for promotion for staff working in this area’) by only 12% of respondents each.

## Discussion

The two-stage online questionnaire has enabled the identification and prioritisation of key areas, from multiple stakeholders, as well as possible barriers and enablers to research across one European country for DER over the next 3–5 years.

### Summary of key findings

Eight broad themes were identified in Stage 1: issues pertaining to the learner; issues pertaining to the educator; working with others in the workplace; workplace culture; curriculum integration; curriculum content; curriculum delivery and assessment/feedback. These themes resulted in 24 priority areas (or items) with no further items identified in Stage 2. The top five ranked priorities were: (1) role of assessments in identifying competence, (2) undergraduate curriculum prepares for practice, (3) promote teamwork within the dental team, (4) role of assessments in identifying underperformance and (5) providing useful feedback. Participants explained why they chose these priorities around seven themes: patient safety; quality of care; investing for the future; policy and political agendas; evidence-based education; improving student learning and personal interest. Using EFA, the items clustered into four overarching factors: teamwork and professionalism (factor 1); measuring and enhancing performance (factor 2); dental workforce issues (factor 3) and curriculum integration and innovation (factor 4). There were small to moderate effect sizes between participant characteristics (including gender, ethnicity, age and professional role) and factor scores. Our study has highlighted primarily individual (eg, motivation, valuing) and institutional (eg, funding, time) barriers and enablers as being of main concern to the conduct of DER.

### The top DER priorities for Scotland for the next 3–5 years compared with existing literature

There were some similarities and differences between our DER priorities and those identified by Palmer and Batchelor^9^ in dentistry and also by Forrest and colleagues^5^ ^10^ in dental hygiene. Similarities between our study and previous research, for example, include: researching dental team working and learning^9^ and curricula being fit for purpose, selection and retention of educators, curriculum innovation and assessment approaches.^5^ However, our findings arguably provide a more comprehensive and granular perspective on DER priorities, going beyond curricula to consider workplace cultures, professionalism, faculty development and technology-enhanced learning. Dental professionals are required to be competent surgeons in an increasingly specialised and complex environment outside of the dental school; hence, these priorities reflect the push for dental training to occur ‘beyond the ‘sheltered’ environment of the dental school’.^25^

There were also many similarities between our DER priorities and those found for MER, and with some notable differences. We directly compare our DER findings with our MER findings from Scotland, as similar methods were used. Broadly speaking, our Stage 1 themes were similar to those of Dennis *et al*.^6^ The items that were identified for DER (and not in MER) were ‘teaching evidence-based practice’ and ‘tailoring teaching to learning needs’, which were ranked 7^th^ and 10^th^, respectively, in Stage 2. One item was split into ‘undergraduate curricula prepare for practice’ and ‘postgraduate curricula prepare for practice’, which were ranked 2^nd^ and 19^th^, respectively. This highlights the importance placed on the undergraduate curriculum in dentistry and dental care due to the sometimes independent practice status at graduation.^26^ For those items added to the DER Stage 2 questionnaire from the MER study, these were ranked highest at 8^th^ and lowest at 22^nd^ highlighting that although some items were not identified in the Stage 1 DER questionnaire, they were considered to be priorities. The top five identified priorities were similar for two priorities (feedback and curricula preparing for practice) but varied for the remaining three with MER spread across a wider range of priorities relating to resilience/well-being, workplace cultures and selection and recruitment. Rather, DER top priorities were focused on teamwork, curriculum and assessment, illustrating participants' concerns for patient safety and the quality of dental care, promoting student learning and investing in the future dental workforce and developing an education evidence base.

Although items in the current study loaded differently onto the factors than for the MER PSE,^6^ this was not unexpected given that we had three additional items on the DER Stage 2 questionnaire to the MER Stage 2 questionnaire and we identified a 4-factor solution for our data (as opposed to the 5-factor solution identified for our earlier MER). However, by examining the meanings intended by the factors there seemed to be more similarities than differences between the factors for the DER and MER questionnaires. Three of the four factors identified in the current study (ie, teamwork and professionalism; measuring and enhancing performance and curriculum integration and innovation) aligned well with four out of five factors identified for our previous MER PSE.^6^ Items grouped under factor 3 for DER (dental workforce issues) were split across two factors in MER (enhancing and valuing the role of educators and building a resilient workforce). The key difference between the two questionnaires was that the item ‘undergraduate curricula prepares for practice’ loaded onto factor 2 ‘measuring and enhancing performance’ factor in DER, whereas it loaded onto the factor ‘curriculum integration and innovation’ in MER. This further highlights a strong focus on assessment and fitness for practice among the dental profession (especially at the undergraduate level), potentially due to dental students' involvement in invasive procedures at an earlier stage.^16^ In certain European countries there is a need for newly graduated dentists to be independent on graduation,^26^ which contrasts with the more structured and lengthier postgraduate training pathways offered in medical education.

### Different stakeholders' priorities compared with previous literature

Participant characteristics (eg, gender, age, ethnicity and professional role) were related to the perceived importance of the four factors. Similar to our MER study,^6^ no regional differences were found, and women rated all factors more highly than men. Unlike in MER, there were no differences found between learners and non-learners, educators and non-educators or researchers and non-researchers. This is potentially due to the smaller dental community where individuals occupy multiple roles. Interestingly, dental care professionals placed greater value on factors 1, 3 and 4 compared with the dentists. This highlights that dentists are particularly concerned with priorities related to assessment and competence relative to dental care professions (ie, nurses, technologists, technicians). This might be due to the higher level of accountability, technical expertise and risk associated with procedures performed by dentists (eg, performing invasive procedures such as tooth drilling) compared with dental care professionals.

### The barriers and enablers to DER compared with existing literature

Common with other studies,^13–15^ our participants identified lack of time, funding and resources as significant barriers to conducting research. It is unsurprising that individuals should feel unmotivated to conduct DER in institutional cultures that do not provide adequate time, funding and institutional resourcing. Interestingly, participants who completed our survey perceived that individuals (ie, peers/colleagues) rather than institutions necessarily were not valuing DER. This mirrors findings in medical education, where medical educators have been argued to have less cultural capital in relation to their clinical research colleagues (citing less respect and financial support); referring to medical education as the ‘Cinderella discipline’.^27^ Similarly, research by Albert and colleagues^28^ identified that the majority of biomedical health researchers exhibited a predominantly negative posture toward social science researchers, where education research arguably fits.^29^

### Methodological challenges and strengths

Unfortunately, inclusion of the patient voice in our study was limited. The response rate for Stage 1 was lower than anticipated, yet there was representation of all the defined stakeholder groups.^30^ Blair and Zinkhan^30^ argue that a theoretical sample with wide diversity is a better quality sample than one with a high response rate but narrow respondents (ie, non-response is not the only criteria for quality when judging a sample). To overcome potential sample bias, we did have an open-ended question in the Stage 2 questionnaire where respondents could add new priorities and barriers/enablers not identified in Stage 1; however, no new topics were identified. This is also important to bear in mind as the sample characteristics varied from Stage 1 to Stage 2, with greater representation in Stage 2 from learners, women and ethnic-minority groups. It was not possible to calculate a response rate for Stage 2 but a large and broad sample of dental education stakeholders across institutions and regions participated.

The recruitment of a large number of participants from a range of stakeholder groups is a strength of the current approach compared with approaches that used the same selected and small sample for all rounds of a Delphi.^5^ ^10^ Furthermore, this type of participatory approach in identifying priorities (as opposed to an expert group setting the priorities ‘top-down’) is thought to promote ownership of research results and stakeholder buy-in,^31^ ^32^ while improving transparency of the provenance of these priorities to funding bodies.^3^ The use of multiple stakeholders alongside our two questionnaires using rankings (rather than Delphi consensus-based methods) and the EFA, enabled less dominant voices (ie, the dental care professionals) to be heard in our research.

### Implications for further research in Scotland and beyond

This PSE provides the necessary first step to developing a national research strategy to focus systematic efforts and to promote DER. There were more similarities than differences in comparing our DER priorities with MER priorities and this highlights areas for synergy and collaboration on important educational issues within and beyond the dental profession. Despite the similarities, there were also differences; therefore it is important for those in other disciplines or countries to conduct PSEs in their own contexts in an order that important contextual features are taken into account and ensuring that limited resources are used wisely. The funding of health professions education research is challenging, hence the need for collaboration and coordination in the presence of limited resources.^1^ We call for individuals and organisations to maximise the enablers and minimise the barriers to DER to promote its flourishing as a discipline. There needs to be systematic efforts to promote DER as a discipline with legitimate career pathways provided through improved resourcing and infrastructure in order to overcome peer stigma, lack of valuing and motivation at an individual level.
