# Supplementary material for: Developing a national dental education research strategy: priorities, barriers and enablers
Source: BMJ Open. 2017 Mar 29;7(3):e013129. doi: 10.1136/bmjopen-2016-013129 (PMC5372062; doi:10.1136/bmjopen-2016-013129)
Supplement: supplementary file [file bmjopen-2016-013129supp.pdf]

Supplementary material: Coding framework showing 'What' themes, subthemes (items), definitions and illustrative quotes

**Overarching Theme**	**Subtheme**	**Item**	**Definition**	**Illustrative quote**
**The learner**	Effectively select/recruit dentists and dental care professionals to reflect needs	4	Relates to how learners are recruited and selected throughout their dental careers to adequately support Scotland's dental care needs	'I know the uni[versity] is now using the system of MMIs [multiple mini interviews] for new dental applicants, but is the system any better than the old system of a formal interview? I have my doubts!' (Female, Clinical Supervisor)
	Understanding the factors that influence career choice	5	Examines issues such as recruiting learners for specific specialities in Scotland and the role that issues such as gender, generation, educational opportunities and culture might play	Not identified in DER Stage 1
	Understanding how to promote resiliency and well-being	6	Understanding how to promote resiliency and well-being in learners as they progress through their training	Not identified in DER Stage 1
**The educator**	Understanding how to better select/approve educators	7	Addresses concerns about the importance of hiring educators who have expressed interest in teaching and also have experience	Not identified in DER Stage 1
	Understanding how to support/value the role of educators	8	Encompasses the many aspects of support an individual needs in order to successfully work as an educator. These may include abstract support such as valuing the role of educators or more concrete aspects of support such as ensuring protected teaching time	Not identified in DER Stage 1
	Understanding how to facilitate faculty development	9	Relates to the idea that it is essential to ensure that educators are given opportunities to develop their skills, knowledge, behaviours and practices as educators	'The quality of the dental work force is directly affected by the quality of the educators (at all levels – UG, PG and continuing). This has implications for patient care. We need further research into how best to 'educate the educators'.' (Female, Dental Education Researcher)
**Working with others in the workplace environment**	Understanding how to promote teamwork	12	Concerns the challenges that must be negotiated by dental health care teams in order to effectively and efficiently work together	'It is important that everyone works together as part of a team.' (Female, Clinical Supervisor)
	Understanding how we foster interprofessionalism	13	The need to address the difficulties and challenges to staff of working within a dental setting in which many inter-professionals must work together as a team	'Introduction of direct access to Dental Therapists. How will this change dental education and impact on employment.' (Male, Clinical Lecturer)
	Understanding how leadership should be developed	14	Concerns teaching and promoting leadership skills as an important aspect of effective team-working	Not identified in DER Stage 1
**Culture of workplace learning**	Understanding how to balance education and service conflicts	10	Concerns the pressures that are perceived to exist between the delivery of service to patients and the provision of education	'Less patients with complex needs being treated at undergraduate level due to general improvement in dental health.' (Male, Clinical Supervisor)

**environments**	Creating an effective workplace learning culture	11	Concerns issues around the workplace learning environment and exploring how the effectiveness of that environment can be enhanced	'What influences the clinical behaviours of young dentists upon graduating from dental school.' (Male, Clinical Lecturer)
**Priorities focussed on curricular integration**	Understanding the roles of the informal/formal curricula	17	The formal curriculum is the explicitly stated and formally offered aspect of a curriculum, whereas the informal curriculum is the often impromptu learning that takes place between learners and educators [1]. This sub-theme refers to the importance of the informal curriculum in dental education as compared to focusing solely on formal curricula	'Linking what happens in the dental school re[garding] teaching with what they get in outreach with non-University employed staff.' (Male, Professor of Oral Surgery)
	Vertically integrating undergraduate and postgraduate curricula	18	Concerns considering dental education as a lengthy continuum and ensuring that undergraduate study is appropriately linked to early and later years of postgraduate training and beyond (e.g. Continuous Professional Development)	'Improving the transition of education (clinical and academic) between undergraduate education (dental school and outreach) and postgraduate education.' (Male, Clinical Lecturer)
**Priorities focussed on curricular content**	Understanding how to enhance communication skills	21	Relates to developing communication skills in learners with patients and colleagues; thought to be important aspects of being a successful dentist or dental care professional	'From the proceedings in the General Dental Council, it is evident that failures in communication form a key common thread in a patient becoming dissatisfied with their care from a dentist. Though students must achieve academic excellence to enter dentistry, it is possible that "softer" skills can be lacking. We must have robust methods to ensure our students are equip[ped] with these skills, if they are going to meet the expectations of the public and the regulator.' (Male, Clinical Lecturer)
	Understanding how professionalism develops	22	This concerns issues that surround teaching, learning, and assessing professionalism, and specifically aims to develop professionalism in learners as they move through their training	'Identifying methods for both teaching and assessment of professionalism and related elements of the curriculum.' (Male, Head of Dental School)
	Ensuring the undergraduate curriculum prepares for practice / is fit for purpose.	15	Relates to the idea of ensuring the current curriculum adequately prepares undergraduate learners for autonomous practice. It includes understanding how best to ensure students have experience of procedures/clinical skills e.g. in relation to reduced contacts with patients for specific procedures due to improvements in dental care	'Potential significant negative impact on student's clinical experience which may lead to problems postgrad[uate] (e.g. increased unnecessary referrals to specialist centres for what should be routine procedures).' (Male, Clinical Lecturer)
	Ensuring the postgraduate curriculum prepares for practice / is fit for purpose	16	Relates to the idea of ensuring the current curriculum adequately prepares learners for practice and is fit for purpose.	'The utilisation of workplace based assessments in postgraduate training posts' (Female, Specialty Trainee)
	Understanding how we teach evidence-based practice	24	Includes the education of evidence-based practice and research	'How should we teach evidence based practice in such a way as to get translation to the clinical setting and achieve the holy grail of getting dentists to use it in their practising lives after they have left dental school. This includes dental therapy and hygiene students as well as

				dental students.’ (Female, Senior Clinical Lecturer)
**Priorities focussed on curricular delivery**	Understanding how technology impacts dental education	19	This topic concerns the idea that dental education is able to successfully incorporate new dental technologies and technology-enhanced learning in their curricula	‘Use of technology to enhance quality and delivery of dental education.’ (Male, Senior Clinical University Teacher)
	Understanding the role of simulation in education	20	This relates to the idea of examining and exploring how simulation should be integrated into the current curriculum and looking at issues such as the impact of simulation-based education on long-term improvements	‘Assessment of the role and effectiveness of simulation techniques in dental education.’ (Male, Head of Dental School)
	Tailoring teaching to individual learning needs	23	This relates to how best to educate learners in specific areas and the most effective teaching methods, including: tailoring teaching needs to individuals’ learning needs; understanding the effect of internationalisation on teaching methods; and the role of peer-assisted learning	‘Methods of tailoring teaching to suit the needs and maximise the success of all types of student.’ (Male, Senior Clinical University Teacher)
**Priorities focussed on assessment and feedback**	Better understanding the role of consistent and robust assessments/ assessment approaches	1	There have been concerns raised about the value of assessments, especially workplace-based assessments. Stakeholders are calling for more evidence surrounding these assessments and understanding of how they can be implemented more successfully	‘Benefits of different forms of assessment- changing demands on dental students and different things they are being asked to do.’ (Male, Senior Dental Officer)
	Understanding how to provide more useful feedback	2	This relates to understanding how to successfully give feedback to learners that is useful, but also examining how learners respond to and utilise feedback	‘Feedback – Good feedback encourages life-long learning’ (Female, Speciality Dentist)
	Ensuring assessments lead to identifying underperformance	3	Concerns whether current assessments successfully capture the skills and abilities learners will need in order to be fit to practice. It includes the need to standardise marking and assess underperformance and the need for evidence that assessments are capturing whether learners are fit for practice	‘Identifying, supporting and rectifying underperformance’ (Male, Postgraduate Trainer)

All items in the Table were identified in Stage 1 DER PSE and were similar to MER[2] priorities except for those indicated below:

Items 5,6,7,8,14 were not identified in Stage 1 DER PSE but were included in Stage 2 based on the MER PSE. If they were not priorities in dentistry we hypothesized that they would not be positively rated. This also overcame the smaller than anticipated response rates in Stage 1 and enabled comparison.

Items 15 and 16 were split into two items for DER PSE.

Items 23 and 24 were identified in DER PSE and not MER PSE.

1. Hafferty FW. Beyond curriculum reform: confronting medicine's hidden curriculum. Academic Medicine 1998;**73**(4):403-7.
2. Dennis AA, Cleland JA, Johnston P, et al. Exploring stakeholders’ views of medical education research priorities: a national survey. Medical Education 2014;**48**(11):1078-91.

Supplementary material: Coding framework showing 'Why' themes, subthemes (items), definitions and illustrative quotes

Theme	Definition	Illustrative quote
Patient safety	This is concerned with ensuring patients are safe when treated by dentists and dental care professionals.	'Damage to patients is too easily effected when Oral Surgery techniques are being developed on live subjects.' (Male, Senior Lecturer)
Quality of Care	This is concerned with the quality of dental care provided.	'Many GDPs do not provide adequate dental care' (Male, Consultant Orthodontist)
Investing in the Future	This is concerned with the idea that certain priorities need to be addressed in order for Dental Education and the NHS to be successful in the future and the concept that by focusing on students, we are investing in the future.	'If the course is adapted to the needs of the population we will be producing the best practitioners.' (Male, Chair in Oral Sciences)
Policy and political agenda	This is concerned with being a part of the larger policy or political agenda for dental education and practice.	'Developing and evaluating graduate attributes. GA is a topic of importance both from a university perspective, as well as for the dental school, particularly as we try to address and deliver a curriculum that complies with the guidelines from the GDC.' (Female, UG Academic Lead)
Evidence–based education	This is concerned with the idea that a reason an issue should be examined further is there is little research evidence to say that a current approach is the best solution or way to proceed.	'I think new innovations are inevitable but are they effective ways of teaching? We possibly don't know but they are expensive to implement' (Female, Senior Clinical Lecturer)
Improved student learning	This is concerned with dental education research being carried out with the aim of improving student learning.	'A quality learning experience for the students - What we do, how we do it and how we can make it better, are the cornerstones of delivering a dynamic curriculum. Currently, its a challenging environment to deliver dental education in, especially with cuts in places, and it is important to maintain and continue to improve the quality of the education we provide.' (Female, Senior Clinical University Teacher)
Personal interest	This is concerned with dental education research being carried out for personal interest.	'Firstly this is my area of interest administratively in the school in which I work.' (Female, Senior Clinical Lecturer)
